# Characterization of CTX-M-14-producing *Escherichia coli* from food-producing animals

**DOI:** 10.3389/fmicb.2015.01136

**Published:** 2015-10-15

**Authors:** Xiao-Ping Liao, Jing Xia, Lei Yang, Liang Li, Jian Sun, Ya-Hong Liu, Hong-Xia Jiang

**Affiliations:** ^1^Laboratory of Veterinary Pharmacology, College of Veterinary Medicine, South China Agricultural UniversityGuangzhou, China; ^2^Jiangsu Co-Innovation Centre for Prevention and Control of Important Animal Infectious Diseases and ZoonosesYangzhou, China

**Keywords:** *Escherichia coli*, CTX-M-14, plasmids, MLST, cephalosporin

## Abstract

Bacterial resistance to the third-generation cephalosporin antibiotics has become a major concern for public health. This study was aimed to determine the characteristics and distribution of *bla*_CTX-M-14_, which encodes an extended-spectrum β-lactamase, in *Escherichia coli* isolated from Guangdong Province, China. A total of 979 *E. coli* isolates isolated from healthy or diseased food-producing animals including swine and avian were examined for *bla*_CTX-M-14_ and then the *bla*_CTX-M-14__-_positive isolates were detected by other resistance determinants [extended-spectrum β-lactamase genes, plasmid-mediated quinolone resistance, *rmtB*, and *floR*] and analyzed by phylogenetic grouping analysis, PCR-based plasmid replicon typing, multilocus sequence typing, and plasmid analysis. The genetic environments of *bla*_CTX-M-14_ were also determined by PCR. The results showed that fourteen CTX-M-14-producing *E. coli* were identified, belonging to groups A (7/14), B1 (4/14), and D (3/14). The most predominant resistance gene was *bla*_TEM_ (*n* = 8), followed by *floR* (*n* = 7), *oqxA* (*n* = 3), *aac(6′)-1b-cr* (*n* = 2), and *rmtB* (*n* = 1). Plasmids carrying *bla*_CTX-M-14_ were classified to IncK, IncHI2, IncHI1, IncN, IncFIB, IncF or IncI1, ranged from about 30 to 200 kb, and with insertion sequence of IS*Ecp1*, IS*26*, or ORF513 located upstream and IS*903* downstream of *bla*_CTX-M-14_. The result of multilocus sequence typing showed that 14 isolates had 11 STs, and the 11 STs belonged to five groups. Many of the identified sequence types are reported to be common in *E. coli* isolates associated with extraintestinal infections in humans, suggesting possible transmission of *bla*_CTX-M-14_ between animals and humans. The difference in the flanking sequences of *bla*_CTX-M-14_ between the 2009 isolates and the early ones suggests that the resistance gene context continues to evolve in *E. coli* of food producing animals.

## Introduction

Enterobacteria with resistance to third and fourth generation cephalosporin antibiotics, especially *Escherichia coli* bearing extended-spectrum β-lactamase genes (ESBLs), have been detected in a wide range of food-producing animals. In 1989, CTX-M-type β-lactamases, whose carriers are highly resistant to cefotaxime but sometimes susceptible to ceftazidime, were considered as a novel group of class-A β-lactamases with extended-spectrum properties. This family of enzymes are well inhibited by clavulanate and tazobactam (Tzouvelekis et al., 2000). Since then, the CTX-M family of ESBLs has become prominent and is common in *E. coli* with many infections occurring in human patients in the community (Livermore and Hawkey, 2005). In addition, the occurrence and prevalence of CTX-M in food-producing animals were also reported frequently (Hammerum et al., 2012; Reich et al., 2013). Rapid dissemination of *bla*_CTX-M_ genes involved plasmids and mobile genetic elements as well as epidemic spread of particular strains (Eckert et al., 2006). IS*Ecp1*-like insertion sequences (ISs) have repeatedly been observed upstream of open reading frames (ORFs) encoding members belonging to the CTX-M-1, CTX-M-2, and CTX-M-9 clusters. ISs such as IS*10*, IS*26*, and IS*903* have also been observed flanking the ORF region of *bla*_CTX-M_ genes (Arduino et al., 2002).

Of the CTX-M enzymes, the CTX-M-1 and CTX-M-9 clusters have been the most frequently reported worldwide, and although in some places CTX-M-2 group remains endemic, the emergence of new CTX-M groups (mainly CTX-M-1 and CTX-M-9) has been documented (D’Andrea et al., 2013). In addition, the CTX-M-14 enzyme is, besides CTX-M-9, the most widespread enzyme of the CTX-M-9 group (Valverde et al., 2009), especially in China (Li et al., 2010; Zheng et al., 2012). CTX-M-14 was first isolated from hospital in China in 1997 (Chanawong et al., 2002). It is a member of the CTX-M-9 cluster and differs from *bla*_CTX-M-9_ only by the substitution Ala 231→Val (Ma et al., 2002), and it has spread almost all over the world (Canton and Coque, 2006). Reports showed that IS*Ecp1*, IS*26*, ORF513, IS*903*, and ORF1005 located upstream and downstream of *bla*_CTX-M-14_, respectively, which might have contributed to its widespread transmission (Izumiya et al., 2005; Eckert et al., 2006; Bae et al., 2007; Navarro et al., 2007). Moreover, most of the literature indicates that novel CTX-M enzyme genes were often derived from or recombinated with CTX-M-14-like β-lactamase (Djamdjian et al., 2011; He et al., 2013; Tian et al., 2014), indicating that *bla*_CTX-M_ genes evolve by homologous recombination between members of different groups, especially with *bla*_CTX-M-14_ (Tian et al., 2014).

Due to the importance of *bla*_CTX-M-14_ in antimicrobial resistance and its limited information in food producing animals, we examined the characteristics and distribution of *bla*_CTX-M-14_ in *E. coli* of food-producing animals in China in this study.

## Materials and Methods

### Bacterial Isolates and CTX-M-14 Detection

From 2002 to 2009, a total of 979 *E. coli* isolates, including 455 isolates in 2002, 119 isolates in 2003–2004, 76 isolates in 2007 and 329 isolates in 2009 were isolated from healthy or diseased food-producing animals from Guangdong Province in China. Samples were collected from rectal swabs of healthy animals, or the liver, heart, lung, or muscle samples of diseased or dead animals. Samples were seeded on MacConkey agar at 37°C, and one colony with typical *E. coli* morphology was selected from each sample. Each isolate was from an individual animal. The bacterial strains were identified by classical biochemical methods and confirmed using the API-20E system (bioMérieux). All confirmed *E. coli* isolates were stored at -80°C in the Luria–Bertani broth medium containing 30% glycerol.

Cefotaxime-resistant *E. coli* isolates were selected on MacConkey agar containing cefotaxime at 2 μg/mL and then the *bla*_CTX-M-14_ gene was detected by PCR analysis with the primer described previously (Yu et al., 2007), the primers and PCR programs were listed in Supplementary Table S1. PCR products were directly sequenced, and then made comparison in the GenBank nucleotide database.

### Antimicrobial Susceptibility Testing and Genes Characterization

The minimum inhibitory concentrations (MICs) of quinolones (nalidixic acid), fluoroquinolones (ciprofloxacin, enrofloxacin, and levofloxacin), third-generation cephalosporins (ceftiofur, cefotaxime, and ceftazidime) and other antimicrobials (olaquindox, ampicillin, trimethoprim-sulfamethoxazole, tetracycline, gentamicin, amikacin, chloramphenicol, and florfenicol) in *bla*_CTX-M-14_ positive isolates were determined by the agar dilution method following the CLSI guidelines. The breakpoints for individual antimicrobial were used as recommended by the CLSI (M100-S19), CLSI (Vet01-A4/Vet01-S2), and DANMAP 98 (olaquindox). *E. coli* ATCC25922 was used as a quality control strain. All *bla*_CTX-M-14_ positive isolates were tested for *bla*_CTX-M-1G_, *bla*_CTX-M-2G_, *bla*_CTX-M-8G_, *bla*_TEM,_ and *bla*_SHV_, *bla*_OXA_ and *bla*_CMY -2_ by PCR and then confirmed by sequencing. At the same time, plasmid-mediated quinolone resistance (PMQR) genes (*qnrA, qnrB, qnrC, qnrD, qnrS, qepA, aac(6′)-Ib-cr*, and *oqxA*), *rmtB* and *floR* were also detected. The PCR programs and primer sequences were described previously (Yu et al., 2007; Yue et al., 2008; Veldman et al., 2011; Li et al., 2013; Liu et al., 2013, 2014). The primers and PCR programs were listed in Supplementary Table S1. All PCR products were directly sequenced, and the results were compared with those in the GenBank nucleotide database.

### Clonal Relatedness

A multiplex PCR methodology was employed to assign isolates harboring *bla*_CTX-M-14_ to one of the four phylogenetic groups (A, B1, B2, or D). Primers and methodology have been described previously (Doumith et al., 2012). For isolates carrying *bla*_CTX-M-14_, multilocus sequence typing (MLST) was performed using seven conserved housekeeping genes (*adk, purA, recA, mdh, icd, gyrB*, and *fumC*; Wirth et al., 2006). The internal fragments of all loci were sequenced. Allelic profiles and sequence type (ST) determinations were performed according to the *E. coli* MLST website^1^ scheme. MLST data were analyzed by using the eBURST program (version 3^2^), which assesses the relationship within clonal complexes. The set minimum group allele number was five. The UPGMA method of START program (version 2) was used to construct phylogenetic grouping tree. The genetic distance is 0.1.

### Transfer of the *bla_CTX-M-14_* Gene and Plasmid Analysis

Transferability of the identified *bla*_CTX-M-14_ genes was determined by conjugation using streptomycin-resistant *E. coli* C600 as the recipient strain (Zheng et al., 2012). Transconjugants were selected on MacConkey agar plates supplemented with cefotaxime (2 μg/mL) and streptomycin (2000 μg/mL). For those isolates that failed in conjugation experiments, plasmid DNA was extracted by QIAGENPrep Plasmid Midi Kit (Qiagen, Germany), and electroporated into electrocompetent *E. coli* DH5α (TaKaRa Biotechnology, Dalian, China) using a Gene Pulser apparatus (Biorad Laboratories). Transformants were selected on MacConkey agar plates supplemented with cefotaxime (2 μg/mL). The presence of *bla*_CTX-M-14_ was confirmed by PCR. Plasmids were preliminarily classified according to their incompatibility group by using the PCR-based replicon typing (PBRT) scheme described previously (Carattoli et al., 2005). PFGE with S1 nuclease (TakaRa Biotechnology, Dalian, China) digestion of whole genomic DNA was performed for all 14 transconjugants and transformants as described previously (Barton et al., 1995). After Southern transfer to a Hybond-N^+^ membrane (GE Healthcare, Little Chalfont, UK), the plasmids were probed with the *bla*_CTX-M-9G_ gene (DIG High Prime DNA Labeling and Detection Starter Kit I, Roche Applied Science, Mannheim, Germany).

### Genetic Environment of *bla_CTX-M-14_*

Detection of the ISs including IS*Ecp1*, IS*26*, ORF513, IS*903*, and ORF1005, which are located upstream or downstream of *bla*_CTX-M-14_, were performed by PCR according to the methods in previous reports (Izumiya et al., 2005; Eckert et al., 2006; Bae et al., 2007; Navarro et al., 2007; Barlow et al., 2008).

## Results

### Antimicrobial Susceptibility and Detection of Resistance Genes

Among the 979 *E. coli* isolates surveyed, fourteen isolates harbored *bla*_CTX-M-14_, among which four were isolated from swine in 2002, two from duck in 2005 and 2007, and the other eight were isolated from swine and duck in 2009. All the 14 *bla*_CTX-M-14_-positive isolates were multidrug resistant (resistant to three or more classes of antimicrobials) and all of them were resistant to ampicillin, cefotaxime, ceftiofur, ceftriaxone, florfenicol, tetracycline, kanamycin, and ciprofloxacin. In addition, the resistance of the *bla*_CTX-M-14_-positive isolates to gentamicin and doxycycline were 64 and 86%, respectively (**Table 1**). Among the fourteen isolates harboring *bla*_CTX-M-14_, only one isolate was confirmed as *bla*_CTX-M-14b_-carrying strain, the other thirteen were *bla*_CTX-M-14a_. In addition, the fourteen isolates were also subjected to the detection of ESBLs, PMQR genes and other resistance genes (*rmtB* and *floR*). The most predominant gene was *bla*_TEM_ (*n* = 8), including six *bla*_TEM-1_, one *bla*_TEM-1b_, and one *bla*_TEM-135_, followed by *floR* (*n* = 7), *oqxA* (*n* = 3), *aac(6′)-1b-cr* (*n* = 2), and *rmtB* (*n* = 1) (**Table 1**).

**Table 1:** Characteristics of the 14 *Escherichia coli* isolates carrying *bla*_CTX-M-14._

Isolates	Origin	Year	Drug-resistant spectrum	Group	Resistant genes	MICs (μg/ml)			Replicon typing	Genetic environment
						CTF	CTX	CTR		
ZLP20	Pig	2002	AMP/CTX/CTR/CTF/KAN/DOX/TET/NAL/ENR/CIP/FLF	A	*bla*_CTX-M-14a_, *oqxA, floR*	64	64	64	F, Y	
ZLP20-D			AMP/ CTR/CTF/CTX		*bla*_CTX-M-14a_	32	64	64	F	IS*Ecp1*, IS*903*
ZLP19	Pig	2002	AMP/CTX/CTR/CTF/KAN/DOX/TET/NAL/ENR/CIP/FLF	A	*bla*_CTX-M-14a_, *floR*	64	64	128	F, Y	
ZLP19-D			AMP/ CTX /CTR/CTF		*bla*_CTX-M-14a_	32	64	64	F	IS*Ecp1*, IS*903*
ZLP21	Pig	2002	AMP/CTX/CTR/CTF/KAN/GEN/DOX/TET/NAL/ENR/CIP/FLF	A	*bla*_CTX-M-14a_	32	32	64	HI1, N	
ZLP21-D			AMP/ CTX /CTR/CTF		*bla*_CTX-M-14a_	32	32	32	HI1, N	IS*Ecp1*, IS*903*
ZLP25	Pig	2002	AMP/CTX/CTR/KAN/DOX/TET/NAL/ENR/CIP/FLF	A	*bla*_CTX-M-14a_, *aac(6’)-1b-cr*	64	32	64	HI1, N	
ZLP25-D			AMP/ CTX /CTR/CTF/TET		*bla*_CTX-M-14a_	16	32	16	HI1, N	IS*Ecp1*, IS*903*
HN428	Duck	2005	AMP/CTR/CXT/CTF/KAN/SM/DOX/TET/NAL/ENR/CIP/FLF	D	*bla*_CTX-M-14a_, *bla*_TEM-1_	64	32	64	FIB, FIC, I1, F	
HN428-D			AMP/ CTX /CTR /TET		*bla*_CTX-M-14a_, *bla*_TEM-1_	4	16	8	FIB, F	IS*Ecp1*, IS*903*
a88	Duck	2007	AMP/CTR/CTF/KAN/SM/TET/NAL/ENR/CIP/ FLF	D	*bla*_CTX-M-14a,_ *bla*_TEM-1_	64	32	64	F, Y, K	
a88-D			AMP/ CTX /CTR		*bla*_CTX-M-14a_	4	16	16	F	IS*Ecp1*, IS*903*
14	Duck	2009	AMP/CTX/CTR/CXT/KAN/GEN/DOX/TET/NAL/ENR/CIP/FLF	B1	*bla*_CTX-M-14a__,_ *bla*_CTX-M-79_	128	256	256	I1, K	
14-D			AMP/ CTX /CTR/CTF/TET		*bla*_CTX-M-14a_	128	256	128	K	IS*Ecp1*, IS*903*
16	Duck	2009	AMP/CTF/CTX/CTR/KAN/GEN/DOX/TET/NAL/ENR/CHL/CIP/FLF	B1	*bla*_CTX-M-14a__,_ *bla*_TEM-135_, *oqxA, floR*	64	128	128	HI2, FIB, K	
16-D			AMP/CTF/CTX/CTR/CHL/CIP/FLF		*bla*_CTX-M-14a_	64	128	32	K	ORF513, IS*903*
40	Duck	2009	AMP/CTX/CTR/KAN/GEN/DOX/TET/NAL/ENR/CIP/FLF	A	*bla*_CTX-M-14a_, *bla*_TEM-1_, *oqxA, floR*	16	64	64	HI2, FIA, F, FIB	
40-D			AMP/CTF/CTX/CTR/GEN/FLF		*bla*_CTX-M-14a_, *floR*	16	32	8	HI2, F	IS*Ecp1*, IS*903*
103	Goose	2009	AMP/CTX/CTR/CXT/CTF/KAN/GEN/DOX/TET/NAL/ENR/CIP/FLF	A	*bla*_CTX-M-14b__,_ *bla*_TEM-1,_ *aac(6’)-1b-cr, rmtB*	64	64	128	I1, FIB, F, K	
103-D			AMP/CTF/CTX/CTR/CHL/CIP/FLF		*bla*_CTX-M-14b_, *rmtB*	64	64	64	I1	IS*Ecp1*, ORF513, IS*903*
132	Goose	2009	AMP/CTX/CTF/CTR/KAN/DOX/TET/NAL/ENR/CIP/FLF	B1	*bla*_CTX-M-14a_, *oqxA, floR*	64	256	256	P, F, K	
132-D			AMP/CTX/CTF/CTR		*bla*_CTX-M-14a_	64	128	32	F	IS*903*
156	Pig	2009	AMP/CTF/CTX/CTR/KAN/GEN/DOX/TET/NAL/ENR/CIP/FLF	D	*bla*_CTX-M-14a_, *bla*_TEM-1,_ *floR*	128	64	128	FIA,P, F, K	
156-D			AMP/CTF/CTX/CTR/KAN/GEN/DOX/TET/NAL/ENR/CIP/FLF		*bla*_CTX-M-14a_, *bla*_TEM-1_, *floR*	128	256	128	K	IS*Ecp1*, IS*903*
173	Pig	2009	AMP/CTX/CTF/CTR/CTF/KAN/GEN/DOX/TET/NAL/ENR/CIP/FLF	A	*bla*_CTX-M-14a_, *bla*_TEM-1_, *floR*	64	64	64	FIB, Y, F, K	
173-D			AMP/CTX/CTF/CTR		*bla*_CTX-M-14a_, *floR*	64	64	64	F	*IS26*, IS*Ecp1*, IS*903*
187	Pig	2009	AMP/CTX/CTF/CTR/KAN/GEN/DOX/TET/NAL/ENR/CIP/FLF	B1	*bla*_CTX-M-14a_, *bla*_TEM-1b_	32	64	64	FIB, I1, Y, F, K	
187-D			AMP/CTX/CTF/CTR/KAN/GEN/CIP		*bla*_CTX-M-14a_	16	64	16	I1	IS*903*

*D, corresponding conjugants or transformants; AMP, Ampicillin; CTX, Cefotaxime; CTF, Ceftiofur; CXT, Cefoxitin; CTR, Ceftriaxone; CTZ, Ceftazidime; STR, Streptomycin; GEN, Gentamycin; KAN, Kanamycin; FLF, Florfenicol; TET, Tetracycline; NAL, Nalidixic acid; CIP, Ciprofloxacin; ENR, Enrofloxacin; DOX, Doxycycline.*

### Clonal Relatedness and Transfer of *bla_CTX-M-14_*

**FIGURE 1:**
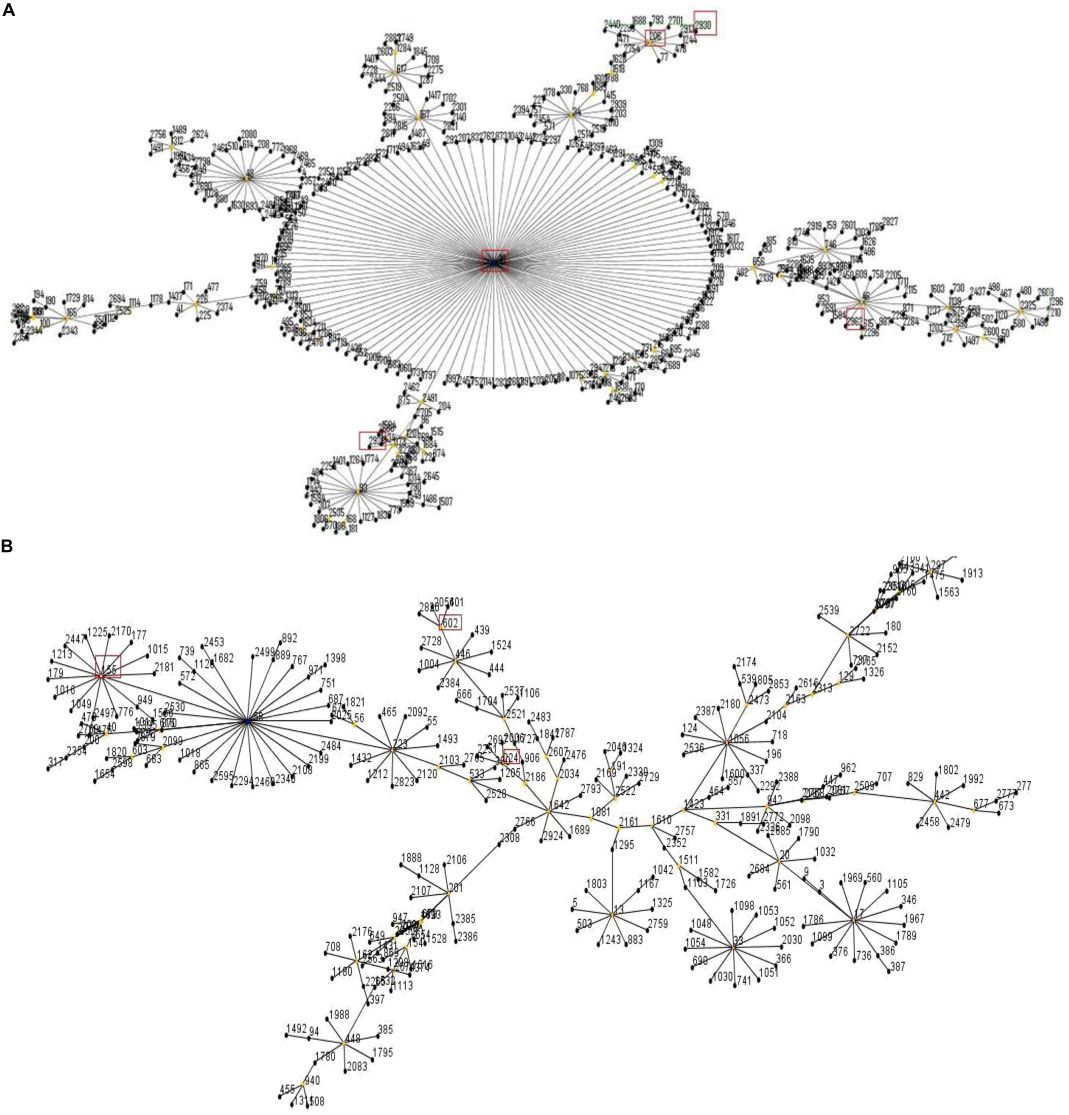
**(A)** eBRUST results of Group 1. The red boxes indicate that the corresponding five sequence types (STs ; ST206, ST10, ST2929, ST2930, and ST2962) we detected in Group 1. **(B)** eBRUST results of Group 2. The red boxes indicate that the corresponding three STs (ST155, ST224, and ST602) we detected in Group 2.

**FIGURE 2:**
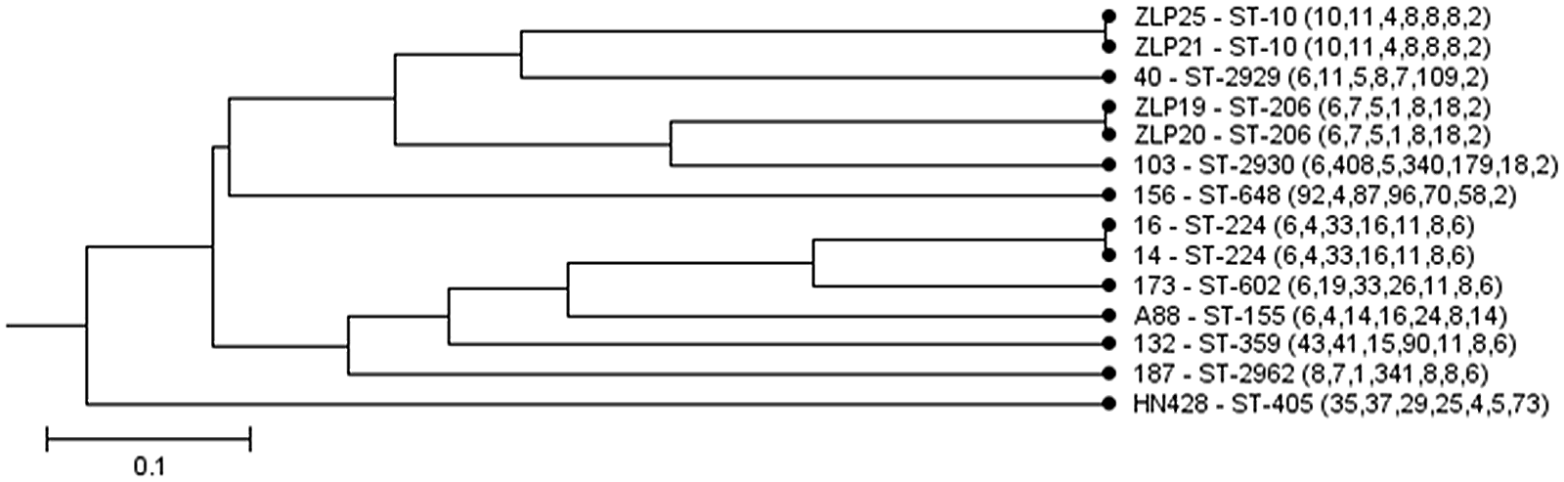
**Evolutionary branches of 14 *bla*_CTX-M-14_-positive *Escherichia Coli***.

Phylogenetic group analysis showed that group A (7/14) was dominant amongst the isolates that produced the CTX-M-14 enzymes, followed by group B1 (4/14) and group D (3/14). None of them belonged to group B2 (**Table 1**). The result of MLST showed that the 14 isolates have 11 ST, among which ST2929 and ST2962 were newly discovered (Supplementary Table S2). The MLST results belonged to five groups. ST10, ST206, ST2929, ST2930, and ST2962 belonged to Group 1 (**Figure 1A**), while ST155, ST224, and ST602 were classified into Group 2 (**Figure 1B**). Furthermore, ST648, ST359, and ST405 belonged to Group14, Group16, and Group17, respectively. Phylogenetic grouping tree suggested that ST10, ST2929, ST206, and ST2930 were close in one branch, while ST224, ST602, ST155, ST359, and ST2962 were clustered in another branch. ST648 and ST405 were separated from others (**Figure 2**). Eight transconjugants and six transformants were successfully obtained by conjugation/transformation experiments. Co-transfer of *bla*_TEM-1_ or *rmtB* or *floR* genes were also detected. The *bla*_CTX-M-14_-positive strain isolated in 2005 co-transferred with *bla*_TEM-1_. Among the 2009 isolates, one had co-transfer of *rmtB*, two had co-transfer of *floR*, and another one had co-transfer of both *bla*_TEM-1_ and *rmtB*. MICs of cefotaxime, ceftiofur, and ceftriaxone increased two–fourfold compared with the recipients.

### Plasmids and Genetic Environment of *bla_CTX-M-14_*

Plasmids containing *bla*_CTX-M-14_ were predominately belonging to IncF (*n* = 5), IncK (*n* = 3), and lncI1 (*n* = 2). Additionally, 2 of the 14 were positive for two replicons (IncHI1 and IncN), another one was positive for IncFIB and IncF, and the remaining one belonged to lncHI2 and lncF. The size of the plasmids ranged from about 30 to 200 kb (Supplementary Figure S2). IS*Ecp1* and IS*903* were found upstream and downstream, respectively, of the *bla*_CTX-M-14_-positive isolates isolated from 2002 to 2007(Supplementary Figure S1). Among the eight positive isolates isolated in 2009, four were detected with IS*Ecp1* and IS*903* upstream and downstream, respectively, one harbored IS*Ecp1* upstream, and the other three carried IS*903* downstream. In addition, IS*26* was confirmed in one strain of 2009, and ORF513 positive isolates were also found in this year. None of the isolates harbored ORF1005.

## Discussion

CTX-M-type ESBLs, with 150 variants, have recently been the most widespread ESBLs in *E. coli*. CTX-M variants can be divided into six clusters: the CTX-M-1, -2, -8, -9, -25, and KLUC groups. Additionally, the most frequently reported groups are CTX-M-1 and CTX-M-9, and CTX-M-14 is the most variant within the latter group (D’Andrea et al., 2013).

In this study, phylogenetic group analysis showed that group A (7/14) was dominant amongst the isolates that produced the CTX-M-14 enzymes, followed by group B1 (4/14) and group D (3/14), which was consistent with the reports in Portugal, Spain, and China (Valverde et al., 2009; Zheng et al., 2012). Previous studies showed that most *E. coli* strains responsible for urinary tract infections and other extraintestinal infections in humans belong to group B2 or, to a lesser extent, to group D (Johnson and Stell, 2000; Yang et al., 2012). Investigation of urinary *E. coli* isolates from 20 widely dispersed tertiary Chinese hospitals revealed although phylogroups D and B2 were most frequently observed, phylogroups A and B1 were also found in *bla*_CTX-M-14_-producing *E. coli* isolates (Cao et al., 2011).

According to recent reports, replicon types of *bla*_CTX-M-14_-positive plasmids belonged to lncF, lncFIB, lncI1, lncA/C, lncN, lncFII, and lncI1-Iγ (Millan et al., 2011; Song et al., 2011; Tamang et al., 2011). In this study, *bla*_CTX-M-14_-carrying plasmids predominately belonged to IncF and IncK. The spread of *bla*_CTX-M-14_ in *E. coli* in Spain is reported to be mediated by IncK plasmids (Valverde et al., 2009), while in Korea and France *bla*_CTX-M-14_ is mostly carried on IncF plasmids (Marcade et al., 2009). IncF plasmids were found frequently to be associated with CTX-M enzyme genes of *E. coli* (Matsumura et al., 2013; Mnif et al., 2013). IncK plasmids may facilitate the ability of *E. coli* to colonize the intestine and, consequently, enhance the pathogenic profile of specific clones or clonal groups (Oshima et al., 2008). Besides, reports showed that the acquisition of IncK plasmids containing *bla*_CTX-M-14_ by group A and B1 *E. coli* clones could have enhanced their ability to colonize the urinary tract in patients exposed to antibiotics (Valverde et al., 2009). IncHI1, IncHI2 and IncN plasmids were rarely reported in *bla*_CTX-M-14_-producing *E. coli*.

In this study, 11 different STs (including two new STs) were detected among 14 *bla*_CTX-M-14_-producing *E. coli* isolates. The findings indicate that no ST predominates in CTX-M-14-producing *E. coli* from food-producing animals of Guangdong. ST10 and ST648 were common in *E. coli* isolated from human and animals (Shabana et al., 2013; Maluta et al., 2014; Xia et al., 2014; Jamborova et al., 2015). ST155 was once found in human, duck, and bovine (Ben Sallem et al., 2012; Sváb et al., 2013; Maluta et al., 2014). ST359 was once reported in human and duck (Maluta et al., 2014). ST405, a global clonal group associated with the global increase of ESBLs, was mainly reported in human origin as well as once reported in rooks and food origins (Jouini et al., 2013; Matsumura et al., 2014; Jamborova et al., 2015). ST602 in *E. coli* of cats was once reported (Nebbia et al., 2014). ST224 was detected in *E. coli* of human, dogs and buffalo origin (Mshana et al., 2011; Dahmen et al., 2013; Aizawa et al., 2014), while ST224 was found in duck origin in 2009 in this study^3^. Moreover, recent reports revealed that *E. coli* of human origin, especially *E. coli*-producing ESBLs associated with urinary tract infection, mainly belonged to the ST10 complex. In Portugal, Spain, and Brazil, ST155 and ST359 were found rising in patients suffering from urinary tract infection (Canton and Coque, 2006). In this study, the STs we have found were mainly reported in human, suggesting that *bla*_CTX-M-14_ could transfer between human and food-producing animals.

Insertion sequences played an important role in the transfer of *bla*_CTX-M-14_. In this study, IS*Ecp1* was detected 42 nucleotides upstream of both *bla*_CTX-M-14a_ and *bla*_CTX-M-14b_. It is of interest to note that an identical 42-bp region has also been detected upstream of different genes encoding ESBLs of the CTX-M-9 cluster, such as CTX-M-9, -14, -16, and -17 (Barlow et al., 2008), which means this subtype may have the same origin as *bla*_CTX-M-14_. From 2002 to 2007, the genetic environment of *bla*_CTX-M-14_-positive isolates was the same, with IS*Ecp1* and IS*903* found upstream and downstream, respectively, while *bla*_CTX-M-14_-positive isolates in 2009 showed diversity of the genetic platform. IS*26* and ORF513 were both found in 2009. It is important to note that ORF513 located upstream of *bla*_CTX-M-14a_ in strain 16-D was the same as *bla*_CTX-M-14b_ of strain 103-D. This showed that resistant genes of incompatible plasmids have the possibility to transfer and then recombine.

Extended-spectrum β-lactamase genes genes were often found to be strongly associated with PMQR or 16S rRNA methyltransferase (16S-RMTase) genes, and some were often found to be located on the same plasmid, both in human and animals (Carattoli, 2009; Liu et al., 2013). In this study, *bla*_CTX-M-14_ of the isolates isolated from 2002 to 2007 tended to conjugate alone, while co-transfer with *bla*_TEM-1_, *rmtB*, or *floR* on the same plasmid were common in the 2009 isolates. Co-existence or co-spread of ESBLs with PMQR, *rmtB* or *floR* suggests that the resistant isolates could be selected by different classes of antibiotics. The fourteen isolates carrying *bla*_CTX-M-14_ were found to be multidrug resistant and showed resistance to more than two non-β-lactam antimicrobial agents, including kanamycin, tetracycline, doxycycline, nalidixic acid, ciprofloxacin, enrofloxacin, and florfenicol. Some of them were also resistant to other cephalosporins, including ceftiofur, cefoxitin, and ceftriaxone, but remained susceptible to ceftazidime fortunately. In addition, although the *bla*_CTX-M-14_-positive isolates showed resistance to kanamycin and gentamycin, most of them (13/14) remain susceptible to amikacin (data not shown), which indicated amikacin might be effective for treating *bla*_CTX-M-14_-positive *E. coli* infection.

## Conclusion

The evolution of *bla*_CTX-M-14_ gradually became diversified in food-producing animals of Guangdong, China, from 2002 to 2009. Findings from this study and previous publications by others suggest that antibiotics, especially the third- and fourth-generation cephalosporins, should be used more prudently in food-producing animals.

## Conflict of Interest Statement

The authors declare that the research was conducted in the absence of any commercial or financial relationships that could be construed as a potential conflict of interest.

## Footnotes

1 http://mlst.warwick.ac.uk/mlst/dbs/Ecoli

2 http://eburst.mlst.net

3 http://mlst.ucc.ie/mlst/dbs/Ecoli/GetTableInfo_html
