# Supplementary material for: Characterization of CTX-M-14-producing Escherichia coli from food-producing animals
Source: Front Microbiol. 2015 Oct 15;6:1136. doi: 10.3389/fmicb.2015.01136 (PMC4606122; doi:10.3389/fmicb.2015.01136)
Supplement: Supplementary file 1 [file Table_1.DOCX]

**Table S1** Primers and programs used for PCR amplification of antimicrobial resistance genes

| Genes | Amplicon size (bp) | Sequence (5'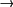3') | PCR programs |
| --- | --- | --- | --- |
| *bla*_CTX-M_ | 543 | F: TTTGCGATGTGCAGTACCAGTAA  R: CGATATCGTTGGTGGTGCCATA | （94℃,5 min）+{（94℃,30s）+（59℃,30s）+（72℃,1min）}×30+（72℃,10min） |
| *bla*_CTX-M-1G_ | 890 | F: ATCCCATGGTTAAAAAATCACTGC  R: CCGTTTCCGCTATTACAAACCGTTG | （94℃,5 min）+{（94℃,45s）+（56℃,45s）+（72℃,1min）}×32+（72℃,10min） |
| *bla*_CTX-M-2G_ | 843 | F: CTCAGAGCATTCGCCGCTCA  R: CCGCCGCAGCCAGAATATCC | （94℃,5 min）+{（94℃,45s）+（56℃,45s）+（72℃,1min）}×32+（72℃,10min） |
| *bla*_CTX-M-8G_ | 947 | F: ACTTCAGCCACACGGATTCA  R: AAGTGGAGCGACAGAGC | （94℃,5 min）+{（94℃,45s）+（56℃,45s）+（72℃,1min）}×32+（72℃,10min） |
| *bla*_CTX-M-9G_ | 876 | F:GCGCATGGTGACAAAGAGAGTGCAA  R: GTTACAGCCCTTCGGCGATGATTC | （94℃,5 min）+{（94℃,45s）+（56℃,45s）+（72℃,1min）}×32+（72℃,10min） |
| *bla*_CMY-2_ | 1143 | F: ATGATGAAAAAATCGTTATGC  R: TTGCAGCTTTTCAAGAATGCG | （94℃,5 min）+{（94℃,1min）+（55℃,45s）+（72℃,90s）}×30+（72℃,10min） |
| *bla*_SHV_ | 885 | F: CACTCA AGGATGTATTGTG  R: TTAGCGTTGCCAGTGCTCG | （94℃,3 min）+{（94℃,30s）+（56℃,30s）+（72℃,1min）}×32+（72℃,10min） |
| *bla*_TEM_ | 1083 | F: ATAAAATTCTTGAAGACGAAA  R: GACAGTTACCAATGCTTAATC | （94℃,5 min）+{（94℃,30s）+（53℃,30s）+（72℃,1min）}×35+（72℃,10min） |
| *bla*_OXA_ | 813 | F: ACACAATACATATCAACTTCGC  R: AGTGTGTTTAGAATGGTGATC | （94℃,3 min）+{（94℃,30s）+（53℃,30s）+（72℃,1min）}×35+（72℃,10min） |
| *qnrA* | 516 | F: ATTTCTCA CGCCAGGATTTG  R: GATCGGCAAAGGTTAGGTCA | （94℃,3 min）+{（94℃,30s）+（53℃,45s）+（72℃,1min）}×32+（72℃,10min） |
| *qnrB* | 469 | F: GATCGTGAAAGCCAGAAAGG  R: ACGATGCCTGGTAGTTGTCC | （94℃,3 min）+{（94℃,30s）+（53℃,45s）+（72℃,1min）}×32+（72℃,10min） |
| *qnrC* | 447 | F: GGGTTGTACATTTATTGAATC  R:TCCACTTTACGAGGTTCT | （94℃,3 min）+{（94℃,45s）+（50℃,45s）+（72℃,1min）}×30+（72℃,10min） |
| *qnrD* | 500～600 | F: CGAGATCAATTTACGGGGAATA  R: AACAAGCTAGAGCGCCTG | （94℃,3 min）+{（94℃,45s）+（50℃,45s）+（72℃,1min）}×30+（72℃,10min） |
| *qnrS* | 417 | F:ACGACATTCGTCAACTGCAA  R: TAAATTGGCACCCTGTAGGC | （94℃,3 min）+{（94℃,45s）+（53℃,45s）+（72℃,1min）}×30+（72℃,10min） |
| *qepA* | 548 | F: CGGCGGCGTGTTGCTGGAGTTCTT  R: CCGACAGGCCCACGACGAGGATGC | （94℃,5 min）+{（94℃,45s）+（60℃,45s）+（72℃,1min）}×30+（72℃,10min） |
| *aac(6′)-Ib-cr* | 482 | F: TTGCGATGCTCTATGAGTGGCTA  R: CTCGAATGCCTGGCGTGTTT | （94℃,5 min）+{（94℃,45s）+（55℃,45s）+（72℃,45s）}×30+（72℃,10min） |
| *oqxA* | 529 | F: AGTCCATACCAACCTCGTCTCC  R: GCGTGGCTTTGAACTCTGC | （94℃,5 min）+{（94℃,30s）+（55℃,30s）+（72℃,30s）}×30+（72℃,10min） |
| *rmtB* | 725 | ACATCAACGATGCCCTCAC  AAGTTCTGTTCCGATGGTC | （94℃,5 min）+{（94℃,30s）+（56℃,30s）+（72℃,30s）}×30+（72℃,10min） |
| *floR* | 480 | TTTGGWCCGCTMTCRGAC  SGAGAARAAGACGAAGAAG | （94℃,5 min）+{（94℃,30s）+（56℃,30s）+（72℃,30s）}×30+（72℃,10min） |

**Table S2** MLST results of *bla*_CTX-M-14_ positive strains

| Strains | Numbers of allele genes | | | | | | | ST | ST clonal complex | Group |
| --- | --- | --- | --- | --- | --- | --- | --- | --- | --- | --- |
|  | *Adk* | *fumC* | *gyrB* | *icd* | *mdh* | *purA* | *recA* |  |  |  |
| ZLP20 | 6 | 7 | 5 | 1 | 8 | 18 | 2 | 206 | ST206 Cplx | 1 |
| ZLP19 | 6 | 7 | 5 | 1 | 8 | 18 | 2 | 206 | ST206 Cplx | 1 |
| ZLP21 | 10 | 11 | 4 | 8 | 8 | 8 | 2 | 10 | ST10 Cplx | 1 |
| ZLP25 | 10 | 11 | 4 | 8 | 8 | 8 | 2 | 10 | ST10 Cplx | 1 |
| HN428 | 35 | 37 | 29 | 25 | 4 | 5 | 73 | 405 | ST405 Cplx | 17 |
| A88 | 6 | 4 | 14 | 16 | 24 | 8 | 14 | 155 | ST155 Cplx | 2 |
| 14 | 6 | 4 | 33 | 16 | 11 | 8 | 6 | 224 | None | 2 |
| 16 | 6 | 4 | 33 | 16 | 11 | 8 | 6 | 224 | None | 2 |
| 40 | 6 | 11 | 5 | 8 | 7 | 109 | 2 | 2929^‡^ | None | 1 |
| 103 | 6 | 408 | 5 | 340^†^ | 179 | 18 | 2 | 2930 | None | 1 |
| 132 | 43 | 41 | 15 | 90 | 11 | 8 | 6 | 359 | None | 16 |
| 156 | 92 | 4 | 87 | 96 | 70 | 58 | 2 | 648 | None | 14 |
| 173 | 6 | 19 | 33 | 26 | 11 | 8 | 6 | 602 | ST446 Cplx | 2 |
| 187 | 8 | 7 | 1 | 341^†^ | 8 | 8 | 6 | 2962^‡^ | None | 1 |

^†^Noval alleles reported in <http://mlst.ucc.ie/mlst/dbs/Ecoli/GetTableInfo_html>

^‡^Noval STs reported in <http://mlst.ucc.ie/mlst/dbs/Ecoli/GetTableInfo_html>

**Figure S1** Genetic environments of the 14 *E. coli* carrying *bla*_CTX-M-14_


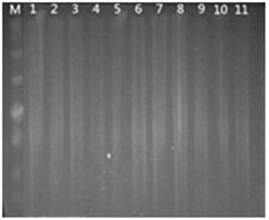

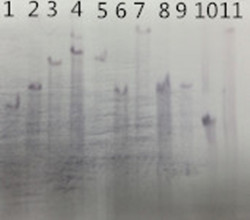


9.42

194.0

145.5

97.0

48.5

23.1

kb

**Figure S2** Plasmids profile of the *bla*_CTX-M-14_ positive strains. Lane M: Low Ranger PFG Marker; Lanes 1-11: 103, 132, 156, 173, 187, ZLP20, ZLP19, ZLP21, ZLP25, a88 and HN428. The other three strains (14, 16 and 40) were not detected.
